# Interaction Between *NOS3* and *HMOX1* on Antihypertensive Drug Responsiveness in Preeclampsia

**DOI:** 10.1055/s-0040-1712484

**Published:** 2020-06-19

**Authors:** Valeria Cristina Sandrim, Marcelo Rizzatti Luizon, Eliane Pilan, Mayara Caldeira-Dias, Fernanda Borchers Coeli-Lacchini, Georgia Kors, Iuly Berndt, Riccardo Lacchini, Ricardo Carvalho Cavalli

**Affiliations:** 1 Department of Pharmacology, Instituto de Biociências de Botucatu da Universidade Estadual Paulista, Botucatu, SP, Brazil; 2 Department of Genetics, Ecology and Evolution, Instituto de Ciências Biológicas da Universidade Federal de Minas Gerais, Belo Horizonte, MG, Brazil; 3 Department of Internal Medicine, Faculdade de Medicina de Ribeirão Preto da Universidade de São Paulo, São Paulo, SP, Brazil; 4 Department of Psychiatric Nursing and Human Sciences, Escola de Enfermagem de Ribeirão Preto, Universidade de São Paulo, Ribeirão Preto, SP, Brazil; 5 Department of Gynecology and Obstetrics, Faculdade de Medicina de Ribeirão Preto, Universidade de São Paulo, Ribeirão Preto, SP, Brazil

**Keywords:** preeclampsia, polymorphism, antioxidant, nitric oxide, pré-eclâmpsia, polimorfismo, antioxidante, óxido nítrico

## Abstract

**Objective** We examined the interaction of polymorphisms in the genes heme oxygenase-1 ( *HMOX1* ) and nitric oxide synthase ( *NOS3* ) in patients with preeclampsia (PE) as well as the responsiveness to methyldopa and to total antihypertensive therapy.

**Methods** The genes *HMOX1* (rs2071746, A/T) and *NOS3* (rs1799983, G/T) were genotyped using TaqMan allele discrimination assays (Applied Biosystems, Foster City, CA, USA ), and the levels of enzyme heme oxygenase-1 *(* HO-1) were measured using enzyme-linked immunosorbent assay (ELISA).

**Results** We found interactions between genotypes of the *HMOX-1* and *NOS3* genes and responsiveness to methyldopa and that PE genotyped as AT presents lower levels of protein HO-1 compared with AA.

**Conclusion** We found interactions between the *HMOX-1* and *NOS3* genes and responsiveness to methyldopa and that the *HMOX1* polymorphism affects the levels of enzyme HO-1 in responsiveness to methyldopa and to total antihypertensive therapy. These data suggest impact of the combination of these two polymorphisms on antihypertensive responsiveness in PE.

## Resumo

**Objetivo** Examinamos a interação dos polimorfismos nos genes heme oxigenase-1 ( *HMOX1* ) eóxido nítrico sintase ( *NOS3* ) em pacientes com pré-eclâmpsia (PE)bem como as capacidades de resposta à metildopa e à terapia anti-hipertensiva.

**Métodos** Os polimorfismos nos genes *HMOX1* (rs2071746, A/T) e *NOS3* (rs1799983, G/T) foram genotipados usando *TaqMan allele discrimination assays* (Applied Biosystems, Foster City, CA, EUA), e os níveis da enzima heme oxigenase-1 (HO-1) foram medidos por *enzyme-linked immunosorbent assay* (ELISA).

**Resultados** Foram encontradas interações entre os genótipos da *HMOX-1* e *NOS3* e responsividade à metildopa, e que pacientes genotipados como AT apresentam níveis mais baixos de proteína HO-1 em comparação com o genótipo AA.

**Conclusão** Foram encontradas interações entre os genes *HMOX-1* e *NOS3* e responsividade à metildopa e que o polimorfismo localizado no gene *HMOX1* afeta os níveis de enzima HO-1 na resposta à metildopa e à terapia anti-hipertensiva. Esses dados sugerem o impacto da combinação desses dois polimorfismos na resposta anti-hipertensiva na PE.

## Introduction

Preeclampsia (PE) is a syndrome characterized by hypertension associated with proteinuria or other systemic signs and is considered one of the major causal factors for maternal and fetal morbidity worldwide. 1 Several studies have explored the role of an unbalance between antioxidant and oxidant agents in the pathophysiology of PE, as reviewed elsewhere. 2 However, despite these efforts, a small number of clinical studies have investigated the heme oxygenase-1 (HO-1), a pivotal enzyme that protects cells against oxidative stress. 3 4 5 6 7 8 Heme oxygenase-1 cleaves heme-producing bilirubin and carbon monoxide (CO), thus promoting cell protection by its antiapoptotic, antioxidant and antiinflammatory properties. Besides, in a rat model of PE a possible effect of HO-1 in regulating blood pressure levels was found. 9 10

The gene code for enzyme HO-1 ( *HMOX1* ) and its GT _n_ polymorphism were related to lower HO-1 expression and associated with non-severe late-onset PE. 11 12 However, other *HMOX1* polymorphisms were not examined in PE, with particular focus on single nucleotide polymorphisms (SNPs) located at the promoter of *HMOX1* . For example, the rs2071746 (A/T) was associated with protective factor for patients with stroke carriers of the A allele, and it was associated with higher expression of *HMOX1.* 13 14

The nuclear factor-erythroid-derived 2-related factor-2 (Nrf2) regulates the expression of several antioxidant proteins, including HO-1. 15 Notably, the rs35652124 T > C polymorphism located at the promoter of nuclear factor, erythroid 2 like 2 ( *NFE2L2* ) gene was found to modulate the forearm vasodilator response in humans. 16 Moreover, the C allele was associated with higher diastolic blood pressure levels in Japanese women, and with high risk of cardiovascular mortality in hemodialysis patients. 17 Notably, Nrf2 binds to the antioxidant response element (ARE) at the *HMOX1* promoter and can regulate HO-1 expression. 15

Remarkably, gene-gene interactions have also been taken into report in pharmacogenomics studies. 18 19 20 Therefore, it is possible that combinations of *NFE2L2* and *HMOX1* genotypes may be associated with the development of PE and with the responsiveness to antihypertensive therapy in patients with PE. In addition, the increased oxidative stress in PE can potentially scavenge and reduce the bioavailability of nitric oxide (NO), which may be impaired by some SNPs of the endothelial nitric oxide synthase ( *NOS3* ) gene. 21 22 23 24 Notably, haplotypes formed by the combination of alleles of *NOS3* polymorphisms were associated with different subgroups of response to antihypertensive therapy in PE. 25

In the present study, we examined the distributions of *NFE2L2* and *HMOX1* polymorphisms in pregnant patients with PE who responded to antihypertensive therapy with those who did not respond to antihypertensive therapy. We further verified whether *NFE2L2* and *HMOX1* polymorphisms affect plasma HO-1 levels in these subgroups of pregnant patients with PE. We also investigated if interactions among *NFE2L2, HMOX1* , and *NOS3* polymorphisms were associated with PE and with the responsiveness to antihypertensive therapy in pregnant patients with PE.

## Methods

### Subjects

Approval for use of human subjects was obtained from the Institutional Review Board at the Ribeirão Preto Medical School of the Universidade de São Paulo (FMRP-USP, in the Portuguese acronym). All pregnant women were enrolled in the High Risk Ambulatory of the University Hospital at the FMRP-USP. Preeclampsia was defined in accordance to the American College of Obstetricians and Gynecologists (ACOG) as high blood pressure (≥ 140 mmHg systolic or ≥ 90 mmHg diastolic at two or more measurements at least 6 h apart) associated with severe features in a woman after 20 weeks of gestation. 1 In the present study, women with preexisting hypertension, with or without superimposed PE were not included.

Written informed consent was provided and maternal venous blood samples were collected. Genomic DNA was isolated from the cellular component of 1 mL of whole blood by a salting-out method and stored at – 20°C until use. Plasma was obtained from centrifugation of whole blood in ethylenediaminetetraacetic acid (EDTA) at 2,000 g for 10 min and stored at – 70°C until assayed.

### Antihypertensive Treatment and Drug Response Evaluation

We carefully monitored for signs and symptoms of PE in pregnant women enrolled in the present study and for fetal surveillance and laboratory tests at least once weekly. Responsiveness to therapy was evaluated through the clinical and laboratory parameters (see below) in response to the antihypertensive drugs treatment. The initial antihypertensive drug was methyldopa (1,000–1,500 mg per day) followed by nifedipine (40–60 mg per day) and/or hydralazine (5–30 mg), which were added in case of lack of significant responses to methyldopa. One of the following clinical and laboratory outcomes were considered to classify a patient as nonresponsive to antihypertensive therapy:

(1) Clinical symptoms including blurred vision, persistent headache or scotomata, persistent right upper quadrant or epigastric pain;
(2) Systolic blood pressure above 140 mmHg and diastolic blood pressure > 90 mmHg, as assessed by the blood pressure curve;
(3) Hemolysis, elevated liver enzymes, low platelet count (HELLP) syndrome; or proteinuria > 2.0 g per 24 h; creatinine > 1.2 mg per 100 mL or blood urea nitrogen > 30 mg per 100 mL; aspartate aminotransferase > 70 Ul ^−1^ and alanine aminotransferase > 60 Ul ^−1^ ; and
(4) Fetal hypoactivity or nonreactive fetus, as revealed by cardio tocography; intrauterine growth restriction, oligoamnio, abnormal biophysical profile score, and Doppler velocimetry abnormalities, as evaluated by ultrasound. 25

In the Supplementary Figure S1 , we show the schematic diagram of the study workflow.

### Genotyping

Genotypes for the rs35652124 polymorphism of the *NFE2L2* gene were determined by polymerase chain reaction-restriction fragment length polymorphism (PCR-RFLP). The forward and reverse primers were respectively: 5′CCTAGAGGAGGTCTCCGTTAG3′ and 5′CTGGTACTATTTTGTGAGTACGTG3′. The PCR reaction generated product of 608 bp that was digested with *Bse* RI (New England Biolabs, Ipswich, MA, USA) restriction enzyme. Three bands were visualized when heterozygote 680 bp, 401 bp, and 268 bp; 2 bands (401 bp and 268 bp when TT genotype), and 1 band 608 bp when CC.

Genotypes for the rs2071746 polymorphism of the *HMOX1* gene were obtained using TaqMan allele discrimination assays (Applied Biosystems, Foster City, CA, USA) using real-time PCR. Probes and primers used were designed by Applied Biosystems (Assay ID: C__15869717_10 for rs2071746). Polymerase chain reactions were performed in a total volume of 12 μl (3 ng of template DNA, 1 × TaqMan genotyping master mix (Life Technologies Corporation, Grand Island, NY, USA) and 1 × TaqMan allele discrimination assay). Thermal cycling was performed in standard conditions, and fluorescence was recorded by the StepOne Plus Real-Time PCR equipment (Applied Biosystems). Results were obtained with manufacturer's software. The two polymorphisms of *NOS3* ( *rs1799983* and *rs2070744* ) were determined using also allele discrimination assays, as described previously. 22

### Enzyme Immunoassays of plasma HO-1

The levels of HO-1 were measured in plasma (EDTA) using a Human total HO-1 */HMOX1* kit (R &D Systems, Minneapolis, MN, USA), according to manufacturer's instructions. Briefly, 50 µL of plasma was used to each patient, and the optical density was determined at 450 nm using in a Synergy 4 microplate reader (BioTek, Winooski, VT, USA).

### Statistical Analysis

The clinical characteristics of groups were compared by Student unpaired *t* -test, Mann-Whitney *U* -test, or χ ^2^ , as appropriate. The modulation of the genotypes for the *NFE2L2* and *HMOX1* polymorphisms on plasma HO-1 concentrations were compared by one-way analysis of variance (ANOVA). The distribution of genotypes was analyzed for deviation from the Hardy-Weinberg equilibrium. The differences in genotype and allele frequencies among subgroups were assessed using the χ ^2^ test. A value of *P* < 0.05 was considered statistically significant.

Multifactor dimensionality reduction (MDR) identifies interactions of genotypes for their ability to classify them into high and low-risk cells or into responsive or nonresponsive groups through cross-validation (CV) steps and permutation testing. 26 We used the robust MDR approach to characterize these interaction models, which performs constructive induction using a Fisher exact test rather than a predetermined threshold and has the advantage of only considering statistically significant genotype combinations in its analysis. 27 The best interaction model was the model that had the maximum testing score and CV consistency. Permutation testing was performed to determine the statistical significance of the best model. 26 28

## Results

Table 1 summarizes the clinical parameters of the pregnant women enrolled in the present study. Preeclampsia was older than healthy pregnant (HP) patients ( *P* < 0.05) and increased body mass index was found in PE compared with HP patients ( *P* < 0.05). We found lower gestational age at delivery (GAD) and lower newborn weights in PE (all *p* < 0.05) compared with HP patients. Supplementary Table S1 shows clinical characteristics of preeclampsia women responsive or not to methyldopa or total antihypertensive therapy. Unresponsiveness (both methyldopa and total therapy) was associated with higher systolic and diastolic blood pressure, and lower GAD and newborn weighs (all *P* < 0.05).

**Table 1:** Demographic characteristics of study subjects

Parameters	Healthy pregnancy	Preeclampsia	
	( *n* = 217)	( *n* = 181)	*p* -value
Age (years)	26 ± 1	27 ± 1	0.48
Ethnicity (% White)	152 (70)	129 (71)	0.91
Current smokers (%)	17 (8)	16 (9)	0.73
BMI (Kg/m ^2^ )	21 ± 1	28 ± 1	< 0.0001
SBP (mmHg)	110 ± 1	142 ± 2	< 0.0001
DPB (mmHg)	70 ± 1	89 ± 1	< 0.0001
Primiparity (%)	98 (45)	76 (42)	*NS*
Fasting glucose (mg/dl)	70 ± 2	69 ± 2	0.48
Hemoglobin (g/dl)	12 ± 1	12 ± 1	1.00
Hematocrit (%)	37 ± 5	37 ± 4	1.00
Urea (mg/dl)	ND	23 ± 1	–
GAD (weeks)	40 ± 1	36 ± 1	0.0053
Newborn weight (g)	3,359 ± 40	2,536 ± 68	< 0.0001
AST (IU/l)	ND	26 ± 17	–
24-hour Pr	ND	1,402 ± 1,628	–
GAS (weeks)	36 ± 1	34 ± 1	0.04

Abbreviations: 24-hour Pr, 24-hour proteinuria; AST, aspartate transaminase; BMI, body mass index; DBP, diastolic blood pressure; GAD, gestational age at delivery; GAS, gestational age at sampling; ND, not determined; SBP, systolic blood pressure.

Values are the mean ± SEM * *p* < 0.05 versus healthy pregnant group.

All the polymorphisms showed no deviation from Hardy-Weinberg equilibrium (all *p* > 0.05, data not shown). The *NFE2L2* and *HMOX1* alleles and genotype frequencies distributions are similar between HP and PE patients ( *P* > 0.05) ( Table 2 ).

**Table 2:** Genotype and allele relative frequencies for *NFE2L2* and *HMOX1* polymorphisms in the study groups

Genes and polymorphisms	Genotypes and alleles	HP n (%)	PE n (%)	OR (95% CI)	*p* -value
*NFE2L2*	TT	89 (41)	76 (42)	1.00 (reference)	–
rs35652124	TC	100 (46)	81 (45)	0.933 (0.515–1.688)	0.880
T > C	CC	28 (13)	24 (13)	0.953 (0.395–2.299)	1.000
	T	278 (64)	236 (65)	1.00 (reference)	–
	C	156 (36)	126 (35)	0.957 (0.536–1.709)	1.000
*HMOX1*	AA	56 (26)	49 (27)	1.00 (reference)	–
rs2071746	AT	106 (49)	89 (49)	0.963 (0.493–1.879)	0.963
A > T	TT	54 (25)	43 (24)	0.924 (0.425–2.011)	0.924
	A	226 (52)	188 (52)	1.00 (reference)	–
	T	208 (48)	174 (48)	1.041 (0.597–1.813)	1.000

Abbreviations: *HMOX1* , heme oxygenase-1; HP, healthy pregnancy; NFE2L2, nuclear factor, erythroid 2 like 2; OR, odds ratio; PE, preeclampsia.

We, then, examined the effects of *NFE2L2* and *HMOX1* polymorphisms on the plasma levels of HO-1 in the groups studied. Due to lack of available plasma, we were not able to measure the levels of HO-1 for all subjects enrolled in the study. Therefore, the values are shown for 177 HP and 116 PE patients. We found no significant differences in the levels of HO-1 between *NFE2L2* and *HMOX1* genotypes nor in HP or in PE ( *P* > 0.05). Genotype and allele relative frequencies for *NFE2L2* and *HMOX1* polymorphisms according to responsiveness to methyldopa and to total antihypertensive therapy are shown in Table 3 . Nuclear factor, erythroid 2 like 2 polymorphism had no effects on the responses to methyldopa or to the total antihypertensive therapy. However, the TT genotype of the rs2071746 (A/T) polymorphism of *HMOX1* was more frequent in the pregnant with PE nonresponsive to methyldopa group ( *P* = 0.02) ( Table 3 ). However, we found no association with total antihypertensive therapy responsiveness.

**Table 3:** Genotype and allele relative frequencies for *NFE2L2* and *HMOX1* polymorphisms according to responsiveness to methyldopa or to the total antihypertensive therapy

	Genotype or allele	Methyldopa responsiveness				Antihypertensive therapy responsiveness			
		R n (%)	NR n (%)	OR (95% CI)	*p-* value	R n (%)	NR n (%)	OR (95% CI)	*p* -value
*NFE2L2*	TT	27 (48)	50 (40)	1.00 (reference)	–	48 (47)	30 (37)	1.00 (reference)	–
rs35652124	TC	23 (41)	58 (47)	1.376 (0.760–2.489)	0.365	43 (42)	38 (48)	1.452 (0.798–2.639)	0.229
T > C	CC	6 (11)	16 (13)	1.418 (0.573–3.510)	0.495	11 (11)	11 (15)	1.732 9 (0.712–4.216)	0.265
	T	76 (68)	159 (64)	1.00 (reference)	–	139 (68)	96 (61)	1.00 (reference)	–
	C	36 (32)	92 (36)	1.195 (0.665–2.148)	0.654	67 (32)	61 (39)	1.359 (0.759–2.430)	0.375
*HMOX1*	AA	15 (28)	33 (26)	1.00 (reference)	–	29 (28)	20 (24)	1.00 (reference)	–
rs2071746	AT	34 (61)	55 (44)	0.776 (0.401–1.503)	0.502	53 (52)	36 (45)	1.010 (0.513–1.985)	1.000
A > T	TT	6 (11)	38 (30)	2.937 (1.226–7.033)	0.020	20 (20)	25 (31)	1.808 (0.826–3.958)	0.168
	A	65 (58)	119 (47)	1.00 (reference)	–	108 (54)	76 (47)	1.00 (reference)	–
	T	47 (42)	131 (53)	1.555 (0.890–2.722)	0.156	92 (46)	85 (53)	1.324 (0.759–2.308)	0.396

Abbreviations: CI, confidence interval; *HMOX1* , heme oxygenase 1; *NFE2L2* , nuclear factor, erythroid 2 like 2; NR, non-responsive; OR, odds ratio; R, responsive.

**Fig. 1:**
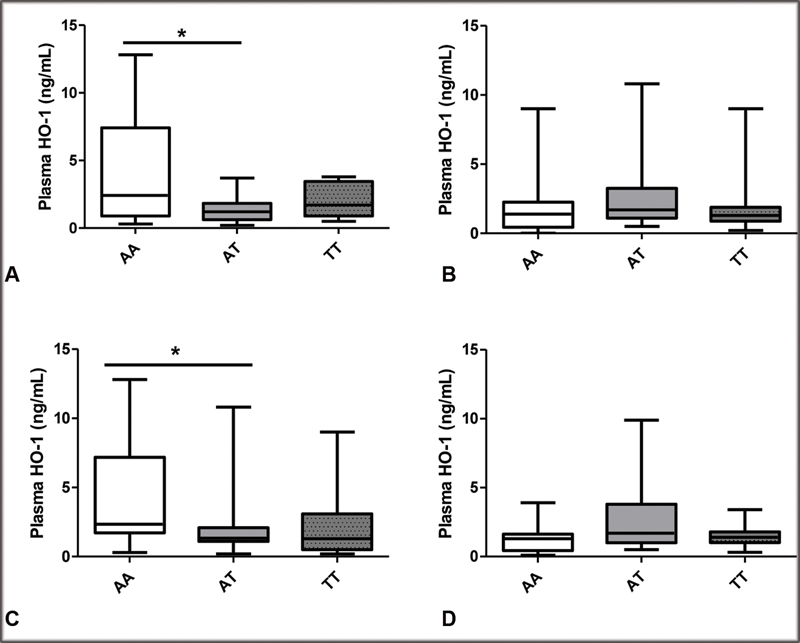
Plasma HO-1 levels in patients with preeclampsia grouped according to the genotypes for the *HMOX1* polymorphism and responsiveness to methyldopa (responsive **A** and nonresponsive **B** ) or total antihypertensive therapy (responsive **C** and nonresponsive **D** ). The bars show the boxplot indicates median [min − max]. * *P* < 0.05 versus the AA genotype.

Regarding the levels of HO-1, we found lower levels of HO-1 in patients with PE patients carrying the genotype AT for the *HMOX1* polymorphism who were responsive to both methyldopa and to the total antihypertensive therapy compared with patients with AA genotype ( Fig. 1 ) ( *P* < 0.05). However, genotypes for the *NFE2L2* polymorphism were not found to be associated with the levels of HO-1 in HP and PE patients, ( Supplementary Figure S2 ) ( *P* > 0.05) neither in responsiveness and *NFE2L2* polymorphism ( Supplementary Figure S3 ) ( *P* > 0.05).

Next, we examined whether interactions among *NFE2L2, HMOX1* , and *NOS3* polymorphisms were associated with PE and with responsiveness to methyldopa and total antihypertensive therapy. Subjects with any missing genotype data for these polymorphisms were not considered in the interaction analyses. We found a significant model of interaction among *HMOX1* and *NOS3* genotypes associated with responsiveness to methyldopa in pregnant patients with PE ( *p* = 0.0125) ( Table 4 ).

**Table 4:** Robust multifactor dimensionality reduction interaction model among the *NFE2L2* , *HMOX1* , and *NOS3* polymorphisms in preeclampsia patients classified as nonresponsive and responsive to methyldopa

Interaction models	Training score	Testing score	CVC	*p* -value
*NOS3* rs1799983	0.7220	0.7220	10/10	–
*HMOX1* rs2071746; *NOS3* rs1799983	0.7186	0.6898	7/10	0.0125 *
*HMOX1* rs2071746; *NOS3* rs2070744 *; NOS3* rs1799983	0.7183	0.6263	6/10	0.1565
*NFE2L2* rs35652124; *HMOX1* rs2071746; *NOS3* rs2070744 *; NOS3* rs1799983	0.5799	0.4572	10/10	0.9420

Abbreviations: CVC, cross-validation consistency; GH, gestational hypertension; *HMOX1* , heme oxygenase-1; HP, healthy pregnancy; MDR, multifactor dimensionality reduction; *NFE2L2* , nuclear factor, erythroid 2 Like 2; *NOS3,* nitric oxide synthase *3;* PE, preeclampsia.

* *P* -value after 1,000 permutations.

**Fig. 2:**
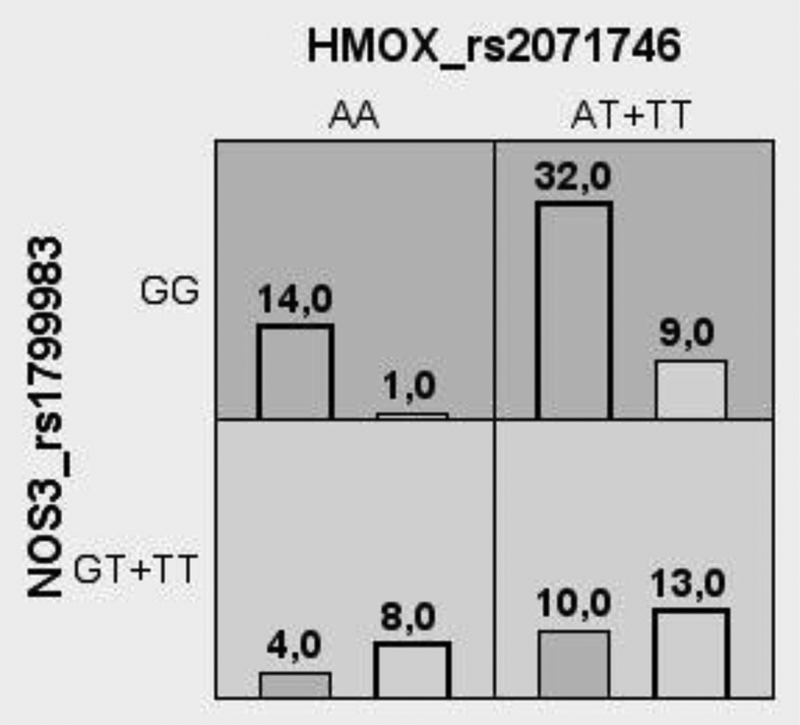
The best robust multifactor dimensionality reduction model of interaction between genotypes for the *HMOX1* rs2071746 A > T and *NOS3* rs1799983 G > T (Glu298Asp) polymorphisms when comparing preeclampsia patients classified according to responsiveness to methyldopa. The distributions of nonresponsive (left bars) and responsive (right bars) patients are illustrated for each combination of multilocus genotypes. The dark gray cells are labeled as high risk or nonresponsive, light gray cells are labeled as low risk or responsive, and white cells are labeled as unknown.

The combinations of genotypes are shown in Fig. 2 . The combinations of the GG genotype for the *NOS3 rs1799983* SNP with the AA and AT + TT genotypes for the *HMOX1* rs2071746SNP were more frequent in the nonresponsive PE patients. Conversely, the combinations of the GT + TT genotypes for the *NOS3 rs1799983* SNP with the AA and AT + TT genotypes for the *HMOX1* rs2071746 A > T SNP were more frequent in the responsive subgroup of PE patients ( Fig. 2 ). However, we found no interactions associated with PE or with responsiveness to total antihypertensive therapy ( Supplementary Tables S2 and S3 ) ( *P* > 0.05).

## Discussion

The present study was the first to examine whether interactions among genes *NFE2L2, HMOX1* , and *NOS3* are associated with PE, and with the responsiveness to methyldopa and total antihypertensive therapy in PE. Moreover, this study was the first to evaluate the effect of *NFE2L2* and *HMOX1* polymorphisms on the plasma levels of HO-1 in both in HP and PE patients and with the responsive and nonresponsive groups to methyldopa and antihypertensive therapy in PE patients. Our main novel findings are (1) the TT genotype of the *HMOX1* rs2071746 polymorphism is associated with the patients with PE who were nonresponsive to methyldopa; (2) the rs2071746 polymorphism affects the plasma levels of HO-1 in the methyldopa and total antihypertensive responsive group of patients with PE, and (3) significant interactions between genotypes of the *HMOX1* rs2071746 and *NOS3* rs1799983 polymorphism were found to be associated with responsiveness to methyldopa in PE patients.

To our knowledge, only two studies have compared the circulating levels of HO-1 in PE and HP patients. One study found higher levels of HO-1 in PE patients. 4 However, we found no significant differences when we compared HP with PE patients. Our findings are supported by another study showing no differences in the serum levels of HO-1 between HP and mild PE women. Moreover, we found no effects of genotypes for *NFE2L2* and *HMOX1* polymorphisms on the plasma levels of HO-1 in PE or in HP patients. 3 While no study has examined whether *NFE2L2* and *HMOX1* polymorphisms affect the responsiveness to methyldopa and total antihypertensive therapy in PE, we have also examined the effects of these polymorphisms on the plasma levels of HO-1 in PE patients. We found that the AT genotype for the rs2071746 polymorphism of *HMOX1* is associated with lower plasma levels of HO-1 in PE patients responsive to methyldopa and total antihypertensive therapy in PE patients. It is probable that we cannot find lower levels of HO-1 in patients with TT genotype due of the lower number of subjects carrying this genotype. However, no functional study was performed to show how the rs2071746 *HMOX1* polymorphism affects *HMOX1* expression.

We found significant interactions among *HMOX1* and *NOS3* polymorphisms associated with responsiveness to methyldopa in PE patients. Although the single-analysis found that the *HMOX1* polymorphism was more frequent in the subgroup of PE patients who are nonresponsive to methyldopa, the combinations with the genotypes for the *NOS3* rs1799983 SNP were associated with both the responsive and the nonresponsive subgroup of PE patients. These findings suggest that specific combinations of genotypes of the *HMOX1* and *NOS3* SNPs may affect the responses to antihypertensive therapy using methyldopa in PE patients.

There is mounting evidence that suggest a relationship between NO and HO-1 pathways. It seems to be a cross-regulation in which NO can be directly involved in the modulation of HO-1 expression, and, likewise, HO-1 expression may increase NO bioavailability. 29 30 Different substances that release NO were shown to significantly upregulate HO-1 mRNA and protein expression, as well as the enzyme activity in different tissues. 31 32 33 In addition, increased endogenous NO derived from stimulated iNOS appears to enhance HO-1 protein expression, which was suppressed in the presence of NOS inhibitors. 34 35 36 These findings show that endogenously generated NO can trigger the expression of HO-1. Nevertheless, the exact molecular mechanisms involving both exogenous and endogenously formed NO (or NO-related species) and how they activate the *HMOX1* gene are not clear. Conversely, there may be other possible mechanisms for vascular NO regulation via HO-1 and its products, as reviewed elsewhere. 30

One possibility is through the modulation of eNOS expression and activity. When the concentrations of l -arginine or BH _4_ are low, eNOS activity can be altered due to eNOS uncoupling, which can generate superoxide (O _2_^−^ ). Superoxide can react spontaneously with NO, leading to the formation of peroxynitrite (ONOO ^−^ ), which, in turn, decreases NO bioavailability. 37 Increased HO-1 expression via pharmacological Nrf2 activation was shown to down-regulate eNOS expression, thereby contributing to eNOS coupling by ensuring stoichiometric balance between BH _4_ and eNOS. 38 Other possible mechanism for the regulation via HO-1 and its products is reducing NO inactivation by inhibiting the sources of O _2_^−^ production, such as NADPH oxidase, or up-regulating antioxidant enzymes, such as superoxide dismutase (SOD) and catalase. 39 40 41 42 43 44 45 Finally, other possible mechanism is compensating the loss of NO by CO effects. Since CO and NO have similar properties, as CO has been shown to reduce vasoconstriction and stimulate vascular relaxation by soluble guanylate cyclase (sGC) and cyclic guanosine monophosphate (cGMP). 33 46 In conclusion, although there are several findings evidencing the relationship between NO and HO-1, further studies need to be performed to fully elucidate the potential mechanisms underlying this cross-regulation.

## Conclusion

In conclusion, the rs2071746 polymorphism of *HMOX1* affects the plasma levels of HO-1 in patients with PE who are responsive to methyldopa and total antihypertensive therapy, and we found significant interactions between the genotypes of *HMOX-1 r* s2071746 and *NOS3* rs1799983 polymorphisms associated with responsiveness to methyldopa. Taken together, our findings suggest that the *HMOX1* and *NOS3* polymorphisms may affect the levels of HO-1 and NO mainly in PE patients who are responsive to methyldopa.

## References

[1] American College of Obstetricians and Gynecologists Task Force on Hypertension in Pregnancy Hypertension in pregnancy. Report of the American College of Obstetricians and Gynecologists' Task Force on Hypertension in Pregnancy Obstet Gynecol 2013 122 05 1122 1131. Doi: 10.1097/01.AOG.0000437382.03963.88 24150027 10.1097/01.AOG.0000437382.03963.88

[2] Sánchez-Aranguren L C Prada C E Riaño-Medina C E Lopez M Endothelial dysfunction and preeclampsia: role of oxidative stress Front Physiol 2014 5 372. Doi: 10.3389/fphys.2014.00372 25346691 10.3389/fphys.2014.00372 PMC4193194

[3] Vitoratos N Papakonstantinou K Deliveliotou A Economou E Panoulis C Hassiakos D Creatsas G K Antepartum and postpartum serum heme oxygenase-1 levels in preeclamptic and normotensive pregnant women In Vivo 2011 25 03 445 450 21576421

[4] Eide I P Isaksen C V Salvesen K A Langaas M Schønberg S A Austgulen R Decidual expression and maternal serum levels of heme oxygenase 1 are increased in pre-eclampsia Acta Obstet Gynecol Scand 2008 87 03 272 279. Doi: 10.1080/00016340701763015 18307065 10.1080/00016340701763015

[5] Tong S Kaitu'u-Lino T J Onda K Beard S Hastie R Binder N K Heme oxygenase-1 is not decreased in preeclamptic placenta and does not negatively regulate placental soluble fms-like tyrosine kinase-1 or soluble endoglin secretion Hypertension 2015 66 05 1073 1081. Doi: 10.1161/HYPERTENSIONAHA.115.05847 26324507 10.1161/HYPERTENSIONAHA.115.05847

[6] Ehsanipoor R M Fortson W Fitzmaurice L E Liao W X Wing D A Chen D B Chan K Nitric oxide and carbon monoxide production and metabolism in preeclampsia Reprod Sci 2013 20 05 542 548. Doi: 10.1177/1933719112459231 23012314 10.1177/1933719112459231 PMC3713541

[7] Farina A Sekizawa A De Sanctis P Purwosunu Y Okai T Cha D H Gene expression in chorionic villous samples at 11 weeks' gestation from women destined to develop preeclampsia Prenat Diagn 2008 28 10 956 961. Doi: 10.1002/pd.2109 18792924 10.1002/pd.2109

[8] McLaughlin B E Lash G E Smith G N Marks G S Nakatsu K Graham C H Brien J F Heme oxygenase expression in selected regions of term human placenta Exp Biol Med (Maywood) 2003 228 05 564 567. Doi: 10.1177/15353702-0322805-28 12709587 10.1177/15353702-0322805-28

[9] Ahmed A Molecular mechanisms and therapeutic implications of the carbon monoxide/hmox1 and the hydrogen sulfide/CSE pathways in the prevention of pre-eclampsia and fetal growth restriction Pregnancy Hypertens 2014 4 03 243 244. Doi: 10.1016/j.preghy.2014.04.013 10.1016/j.preghy.2014.04.013 26104642

[10] George E M Cockrell K Aranay M Csongradi E Stec D E Granger J P Induction of heme oxygenase 1 attenuates placental ischemia-induced hypertension Hypertension 2011 57 05 941 948. Doi: 10.1161/HYPERTENSIONAHA.111.169755 21383306 10.1161/HYPERTENSIONAHA.111.169755 PMC3085942

[11] Chen Y H Lin S J Lin M W Tsai H L Kuo S S Chen J W Microsatellite polymorphism in promoter of heme oxygenase-1 gene is associated with susceptibility to coronary artery disease in type 2 diabetic patients Hum Genet 2002 111 01 1 8. Doi: 10.1007/s00439-002-0769-4 12136229 10.1007/s00439-002-0769-4

[12] Kaartokallio T Klemetti M M Timonen A Uotila J Heinonen S Kajantie E Microsatellite polymorphism in the heme oxygenase-1 promoter is associated with nonsevere and late-onset preeclampsia Hypertension 2014 64 01 172 177. Doi: 10.1161/HYPERTENSIONAHA.114.03337 24799610 10.1161/HYPERTENSIONAHA.114.03337

[13] Cao L Zhang Z Cai B Bai W Zhang Y Sun W Association of heme oxygenase-1 gene rs2071746 polymorphism with vascular outcomes in patients with atherosclerotic stroke J Neurol Sci 2014 344(1-2):154 157 25016572 10.1016/j.jns.2014.06.046

[14] Ono K Goto Y Takagi S Baba S Tago N Nonogi H Iwai N A promoter variant of the heme oxygenase-1 gene may reduce the incidence of ischemic heart disease in Japanese Atherosclerosis 2004 173 02 315 319. Doi: 10.1016/j.atherosclerosis.2003.11.021 15064108 10.1016/j.atherosclerosis.2003.11.021

[15] Singh S Vrishni S Singh B K Rahman I Kakkar P Nrf2-ARE stress response mechanism: a control point in oxidative stress-mediated dysfunctions and chronic inflammatory diseases Free Radic Res 2010 44 11 1267 1288. Doi: 10.3109/10715762.2010.507670 20815789 10.3109/10715762.2010.507670

[16] Marczak E D Marzec J Zeldin D C Kleeberger S R Brown N J Pretorius M Lee C R Polymorphisms in the transcription factor NRF2 and forearm vasodilator responses in humans Pharmacogenet Genomics 2012 22 08 620 628. Doi: 10.1097/FPC.0b013e32835516e5 22668754 10.1097/FPC.0b013e32835516e5 PMC3599320

[17] Shimoyama Y Mitsuda Y Tsuruta Y Hamajima N Niwa T Polymorphism of Nrf2, an antioxidative gene, is associated with blood pressure and cardiovascular mortality in hemodialysis patients Int J Med Sci 2014 11 07 726 731. Doi: 10.7150/ijms.8590 24904228 10.7150/ijms.8590 PMC4045792

[18] Pander J Wessels J A Mathijssen R H Gelderblom H Guchelaar H J Pharmacogenetics of tomorrow: the 1 + 1 = 3 principle Pharmacogenomics 2010 11 07 1011 1017. Doi: 10.2217/pgs.10.87 20602619 10.2217/pgs.10.87

[19] Luizon M R Palei A CT Belo V A Amaral L M Lacchini R Duarte G Gene-gene interactions in the NAMPT pathway, plasma visfatin/NAMPT levels, and antihypertensive therapy responsiveness in hypertensive disorders of pregnancy Pharmacogenomics J 2017 17 05 427 434. Doi: 10.1038/tpj.2016.35 27168100 10.1038/tpj.2016.35

[20] Luizon M R Pereira D A Sandrim V C Pharmacogenomics of hypertension and preeclampsia: focus on gene-gene interactions Front Pharmacol 2018 9 168. Doi: 10.3389/fphar.2018.00168 29541029 10.3389/fphar.2018.00168 PMC5835759

[21] Sandrim V C Palei A CT Metzger I F Gomes V A Cavalli R C Tanus-Santos J E Nitric oxide formation is inversely related to serum levels of antiangiogenic factors soluble fms-like tyrosine kinase-1 and soluble endogline in preeclampsia Hypertension 2008 52 02 402 407. Doi: 10.1161/HYPERTENSIONAHA.108.115006 18574068 10.1161/HYPERTENSIONAHA.108.115006

[22] Muniz L Luizon M R Palei A CT Lacchini R Duarte G Cavalli R C eNOS tag SNP haplotypes in hypertensive disorders of pregnancy DNA Cell Biol 2012 31 12 1665 1670. Doi: 10.1089/dna.2012.1768 23062210 10.1089/dna.2012.1768

[23] de Miranda J A Lacchini R Belo V A Lanna C M Sertorio J T Luizon M R Tanus-Santos J E The effects of endothelial nitric oxide synthase tagSNPs on nitrite levels and risk of hypertension and obesity in children and adolescents J Hum Hypertens 2015 29 02 109 114. Doi: 10.1038/jhh.2014.48 24943287 10.1038/jhh.2014.48

[24] Metzger I F Luizon M R Lacchini R Ishizawa M H Tanus-Santos J E Effects of endothelial nitric oxide synthase tagSNPs haplotypes on nitrite levels in black subjects Nitric Oxide 2013 28 33 38. Doi: 10.1016/j.niox.2012.10.002 23069892 10.1016/j.niox.2012.10.002

[25] Sandrim V C Palei A CT Luizon M R Izidoro-Toledo T C Cavalli R C Tanus-Santos J E eNOS haplotypes affect the responsiveness to antihypertensive therapy in preeclampsia but not in gestational hypertension Pharmacogenomics J 2010 10 01 40 45. Doi: 10.1038/tpj.2009.38 19704415 10.1038/tpj.2009.38

[26] Motsinger A A Ritchie M D Multifactor dimensionality reduction: an analysis strategy for modelling and detecting gene-gene interactions in human genetics and pharmacogenomics studies Hum Genomics 2006 2 05 318 328. Doi: 10.1186/1479-7364-2-5-318 16595076 10.1186/1479-7364-2-5-318 PMC3500181

[27] Gui J Andrew A S Andrews P Nelson H M Kelsey K T Karagas M R Moore J H A robust multifactor dimensionality reduction method for detecting gene-gene interactions with application to the genetic analysis of bladder cancer susceptibility Ann Hum Genet 2011 75 01 20 28. Doi: 10.1111/j.1469-1809.2010.00624.x 21091664 10.1111/j.1469-1809.2010.00624.x PMC3057873

[28] Luizon M R Palei A CT Sandrim V C Tissue inhibitor of matrix metalloproteinase-1 polymorphism, plasma TIMP-1 levels, and antihypertensive therapy responsiveness in hypertensive disorders of pregnancy Pharmacogenomics J 2014 14 06 535 541. Doi: 10.1038/tpj.2014.26 24913092 10.1038/tpj.2014.26

[29] Motterlini R Green C J Foresti R Regulation of heme oxygenase-1 by redox signals involving nitric oxide Antioxid Redox Signal 2002 4 04 615 624. Doi: 10.1089/15230860260220111 12230873 10.1089/15230860260220111

[30] Pae H O Son Y Kim N H Jeong H J Chang K C Chung H T Role of heme oxygenase in preserving vascular bioactive NO Nitric Oxide 2010 23 04 251 257. Doi: 10.1016/j.niox.2010.08.002 20713168 10.1016/j.niox.2010.08.002

[31] Foresti R Motterlini R The heme oxygenase pathway and its interaction with nitric oxide in the control of cellular homeostasis Free Radic Res 1999 31 06 459 475. Doi: 10.1080/10715769900301031 10630670 10.1080/10715769900301031

[32] Durante W Kroll M H Christodoulides N Peyton K J Schafer A I Nitric oxide induces heme oxygenase-1 gene expression and carbon monoxide production in vascular smooth muscle cells Circ Res 1997 80 04 557 564. Doi: 10.1161/01.res.80.4.557 9118487 10.1161/01.res.80.4.557

[33] Sammut I A Foresti R Clark J E Exon D J Vesely M J Sarathchandra P Carbon monoxide is a major contributor to the regulation of vascular tone in aortas expressing high levels of haeme oxygenase-1 Br J Pharmacol 1998 125 07 1437 1444. Doi: 10.1038/sj.bjp.0702212 9884071 10.1038/sj.bjp.0702212 PMC1565726

[34] Kitamura Y Furukawa M Matsuoka Y Tooyama I Kimura H Nomura Y Taniguchi T In vitro and in vivo induction of heme oxygenase-1 in rat glial cells: possible involvement of nitric oxide production from inducible nitric oxide synthase Glia 1998 22 02 138 148. Doi: 10.1002/(SICI)1098-1136(199802)22:2<138:AID-GLIA5>3.0.CO;2-3 9537834

[35] Immenschuh S Tan M Ramadori G Nitric oxide mediates the lipopolysaccharide dependent upregulation of the heme oxygenase-1 gene expression in cultured rat Kupffer cells J Hepatol 1999 30 01 61 69. Doi: 10.1016/s0168-8278(99)80008-7 9927151 10.1016/s0168-8278(99)80008-7

[36] Datta P K Lianos E A Nitric oxide induces heme oxygenase-1 gene expression in mesangial cells Kidney Int 1999 55 05 1734 1739. Doi: 10.1046/j.1523-1755.1999.00429.x 10231435 10.1046/j.1523-1755.1999.00429.x

[37] Schulz E Jansen T Wenzel P Daiber A Münzel T Nitric oxide, tetrahydrobiopterin, oxidative stress, and endothelial dysfunction in hypertension Antioxid Redox Signal 2008 10 06 1115 1126. Doi: 10.1089/ars.2007.1989 18321209 10.1089/ars.2007.1989

[38] Heiss E H Schachner D Werner E R Dirsch V M Active NF-E2-related factor (Nrf2) contributes to keep endothelial NO synthase (eNOS) in the coupled state: role of reactive oxygen species (ROS), eNOS, and heme oxygenase (HO-1) levels J Biol Chem 2009 284 46 31579 31586. Doi: 10.1074/jbc.M109.009175 19797052 10.1074/jbc.M109.009175 PMC2797228

[39] Sue Y M Cheng C F Chang C C Chou Y Chen C H Juan S H Antioxidation and anti-inflammation by haem oxygenase-1 contribute to protection by tetramethylpyrazine against gentamicin-induced apoptosis in murine renal tubular cells Nephrol Dial Transplant 2009 24 03 769 777. Doi: 10.1093/ndt/gfn545 18842672 10.1093/ndt/gfn545

[40] Datla S R Dusting G J Mori T A Taylor C J Croft K D Jiang F Induction of heme oxygenase-1 in vivo suppresses NADPH oxidase derived oxidative stress Hypertension 2007 50 04 636 642. Doi: 10.1161/HYPERTENSIONAHA.107.092296 17679649 10.1161/HYPERTENSIONAHA.107.092296

[41] Jiang F Roberts S J Datla Sr Dusting G J NO modulates NADPH oxidase function via heme oxygenase-1 in human endothelial cells Hypertension 2006 48 05 950 957. Doi: 10.1161/01.HYP.0000242336.58387.1f 16982957 10.1161/01.HYP.0000242336.58387.1f

[42] Wang X Wang Y Kim H P Nakahira K Ryter S W Choi A MK Carbon monoxide protects against hyperoxia-induced endothelial cell apoptosis by inhibiting reactive oxygen species formation J Biol Chem 2007 282 03 1718 1726. Doi: 10.1074/jbc.M607610200 17135272 10.1074/jbc.M607610200

[43] Turkseven S Kruger A Mingone C J Kaminski P Inaba M Rodella L F Antioxidant mechanism of heme oxygenase-1 involves an increase in superoxide dismutase and catalase in experimental diabetes Am J Physiol Heart Circ Physiol 2005 289 02 H701 H707. Doi: 10.1152/ajpheart.00024.2005 15821039 10.1152/ajpheart.00024.2005

[44] Kruger A L Peterson S Turkseven S Kaminski P M Zhang F F Quan S D-4F induces heme oxygenase-1 and extracellular superoxide dismutase, decreases endothelial cell sloughing, and improves vascular reactivity in rat model of diabetes Circulation 2005 111 23 3126 3134. Doi: 10.1161/CIRCULATIONAHA.104.517102 15939814 10.1161/CIRCULATIONAHA.104.517102

[45] Ahmad M Zhao X Kelly M R Kandhi S Perez O Abraham N G Wolin M S Heme oxygenase-1 induction modulates hypoxic pulmonary vasoconstriction through upregulation of ecSOD Am J Physiol Heart Circ Physiol 2009 297 04 H1453 H1461. Doi: 10.1152/ajpheart.00315.2009 19666846 10.1152/ajpheart.00315.2009 PMC2770763

[46] Zhang F Kaide J I Rodriguez-Mulero F Abraham N G Nasjletti A Vasoregulatory function of the heme-heme oxygenase-carbon monoxide system Am J Hypertens 2001 14(6 Pt 2):62S 67S 11411767 10.1016/s0895-7061(01)02071-4

