# Supplementary material for: Interaction Between NOS3 and HMOX1 on Antihypertensive Drug Responsiveness in Preeclampsia
Source: Rev Bras Ginecol Obstet. 2020 Jun 19;42(8):460–7. doi: 10.1055/s-0040-1712484 (PMC10309231; doi:10.1055/s-0040-1712484)
Supplement: Supplementary file 1 — Supplementary Material [file 10-1055-s-0040-1712484-s200018.pdf]

Table S1 Demographic characteristics according to responsiveness to methyldopa and total antihypertensive therapy

Parameters	Methyldopa Responsiveness			Antihypertensive Responsiveness		
	Responsive (n = 56)	Nonresponsive (n = 125)	p-value	Responsive (n = 102)	Nonresponsive (n = 79)	p-value
Age (years)	27 ± 1	26 ± 1	0.54	27 ± 1	27 ± 1	1.0
Ethnicity (% White)	39 (70)	87 (70)	0.63	72 (71)	55 (70)	0.88
Current Smoking (%)	6 (10)	11 (9)	0.55	12 (12)	4 (5)	0.11
BMI (Kg/m^2)	29 ± 1	26 ± 1	0.07	29 ± 1	27 ± 1	0.16
SBP (mmHg)	130 ± 1	148 ± 1	<0.0001	135 ± 2	151 ± 2	<0.0001
DPB (mmHg)	81 ± 1	92 ± 1	<0.0001	85 ± 1	94 ± 1	<0.0001
Fasting Glucose (mg/dL)	80 ± 2	79 ± 2	0.76	78 ± 2	81 ± 3	0.39
24-h-Pr (mg/24h)	634 ± 127	1559 ± 191	<0.0001	1087 ± 199	1591 ± 213	0.09
Primiparity (%)	20 (36)	60 (48)	0.57	43 (42)	37 (47)	0.53
GAD (weeks)	39 ± 1	35 ± 1	0.01	38 ± 1	34 ± 1	<0.0001
Newborn weight (g)	3256 ± 81	2245 ± 74	<0.0001	2921 ± 75	1989 ± 91	<0.0001
GAS (weeks)	35 ± 1	34 ± 1	0.54	35 ± 1	33 ± 1	0.16

GH, gestational hypertension; PE, preeclampsia; BMI, body mass index; SBP, systolic blood pressure; DBP, diastolic blood pressure; 24-h-Pr, 24-h proteinuria; GAD, gestational age at delivery; GAS, gestational age at sampling.Values are the mean ± S.E.M

Table S2 Robust MDR interaction model among the *NFE2L2*, *HMOX1* and *NOS3* polymorphisms when PE patients were compared with HP subjects

Interaction Models	Training score	Testing score	CVC	p-value
NOS3 rs2070744	0.5652	0.5469	9/10	–
NFE2L2 rs35652124; *HMOX1* rs2071746	0.5912	0.5620	9/10	0.3620
NFE2L2 rs35652124; *HMOX1* rs2071746; *NOS3* rs207074;	0.6139	0.5207	6/10	0.6795
NFE2L2 rs35652124; *HMOX1* rs2071746; *NOS3* rs2070744; *NOS3* rs1799983	0.6401	0.5807	10/10	0.2235

CVC, cross-validation consistency; GH, gestational hypertension; HP, healthy pregnant; PE, preeclampsia;*NFE2L2*, Nuclear Factor, Erythroid 2 Like 2; *HMOX1*, Hemeoxygenase 1; MDR, Multifactor dimensionality reduction; *p-value after 1.000 permutations

Table S3 Robust MDR interaction model among the *NFE2L2*, *HMOX1* and *NOS3* polymorphisms in PE patients classified as nonresponsive and responsive to total antihypertensive therapy

Interaction Models	Training score	Testing score	CVC	p-value
NOS3 rs1799983	0.6383	0.6288	10/10	–
NOS3 rs2070744; *NOS3* rs1799983	0.6631	0.6321	7/10	0.1155
NFE2L2 rs35652124; *NOS3* rs2070744; *NOS3* rs1799983	0.6692	0.5870	9/10	0.3825
NFE2L2 rs35652124; *HMOX1* rs2071746; *NOS3* rs2070744; *NOS3* rs1799983	0.5412	0.4805	10/10	0.8870

CVC, cross-validation consistency; GH, gestational hypertension; HP, healthy pregnant; PE, preeclampsia; *HMOX1*, Hemeoxygenase 1; MDR, Multifactor dimensionality reduction; *NFE2L2*, Nuclear Factor, Erythroid 2 Like 2; *NOS3*, ….; *p-value after 1.000 permutations

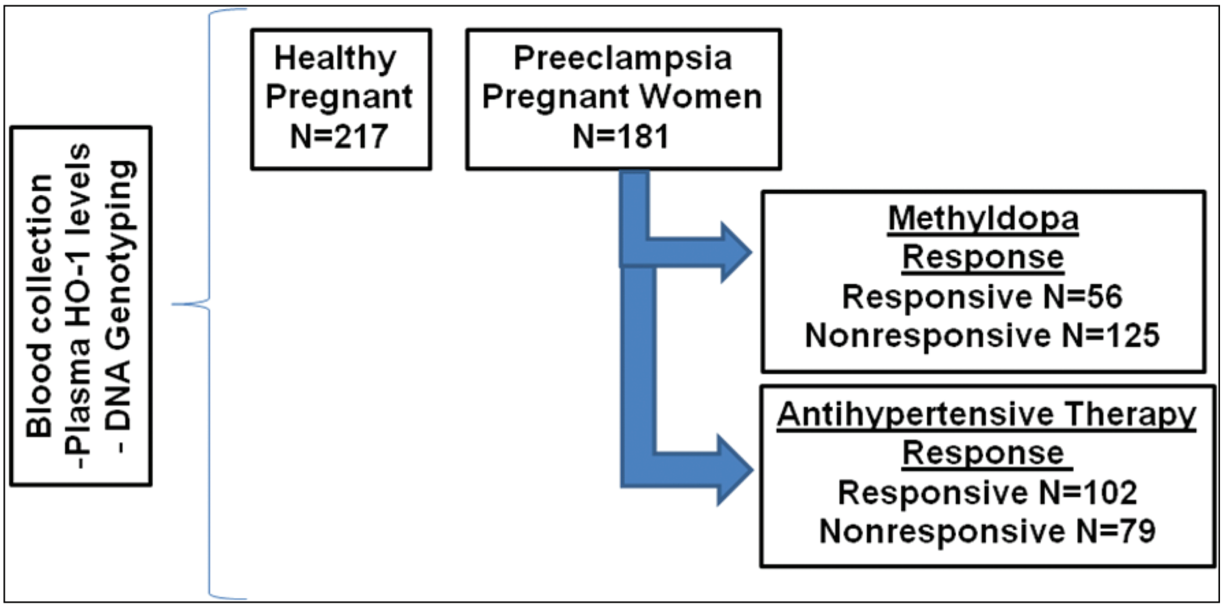

Supplementary Fig. S1 Schematic diagram of the study workflow.

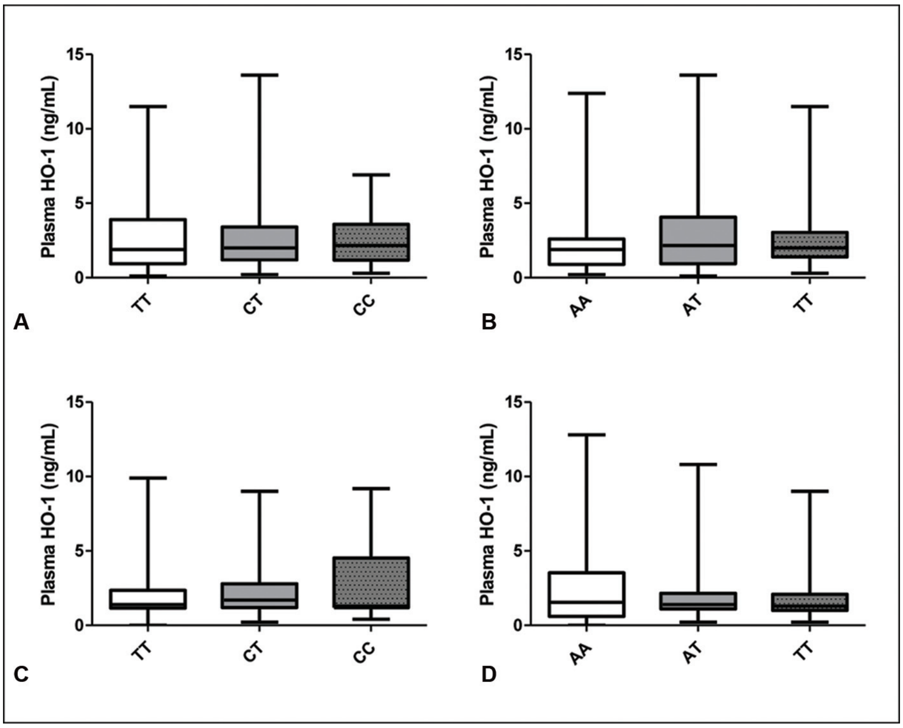

Supplementary Fig. S2 Plasma HO-1 levels in healthy pregnant women (**A** and **B**) and patients with preeclampsia (**C** and **D**) grouped according to the genotypes for the *NFE2L2* polymorphism (A and C) and *HMOX1* polymorphism. The bars show the boxplot indicates median [min – max].

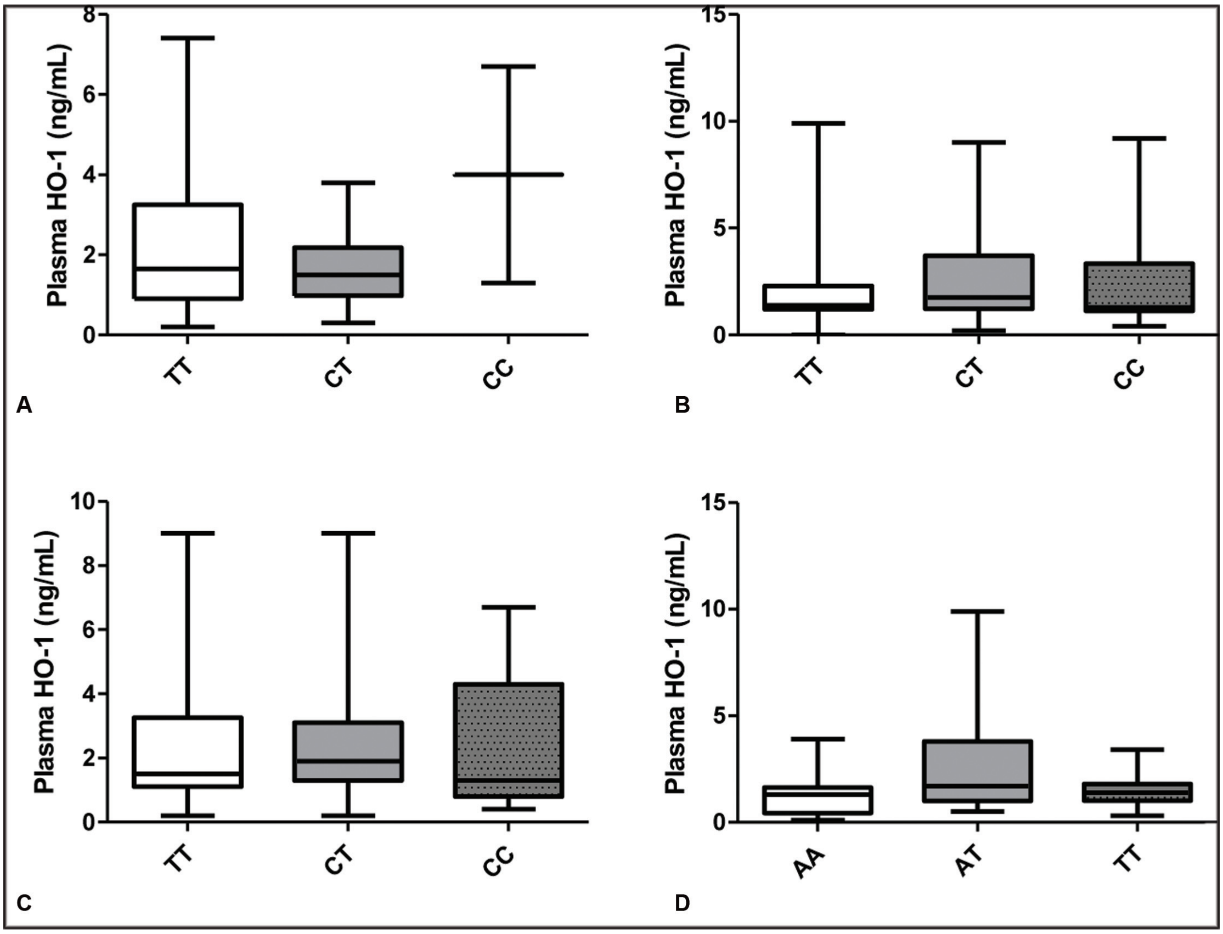

Supplementary Fig. S3 Plasma HO-1 levels in patients with preeclampsia grouped according to the genotypes for the *NFE2L2* polymorphism and responsiveness to methyldopa (responsive **A** and nonresponsive **B**) or total antihypertensive therapy (responsive **C** and nonresponsive **D**). The bars show the box plot indicates median [min – max].
